# The Chemical and Physical Effects of Fillings upon Teeth

**Published:** 1881-09

**Authors:** Thomas Fletcher


### ARTICLE IV.

### *The Chemical and Physical Effects of Fillings Upon Teeth.*

BY THOMAS FLETCHER, F. C. S.

The article on this subject by Dr. Mayr, in the *British Journal of Dental Science*, for May 15th, is one which cannot be allowed to pass without comment. Two things he states can readily be taken for granted. First, that "his practice in Dentistry is very limited," second, that "the task he has undertaken is almost too difficult for him." Any one reading his paper with a knowledge of the subject would at once give him credit for correctness, at least, on these two points. Why he should attempt to do what he has done, knowing so well his own incapacity, is hard to see. It might be desirable to know how he connects "lazy schoolboys" and "algebra to equations in the third degree" with Dentistry, but it will be better to examine at once his positive statements.

First, we will take the remark that "gold *certainly does not* exclude moisture as well as *any* of the other fillings." To any one who has actually tested the moisture tightness of soft gold or of cohesive gold properly packed with ball-faced pluggers, the statement is an absurdity. I am not by any means a first-rate operator, but I will undertake to make a plug of either soft or cohesive gold absolutely tight against penetrating dyes, and any second-rate operator who understands his material and instruments can do the same easily.

If Dr. Mayr eats "oyster stew at a temperature of 130° F.," getting it carefully in contact with his gold fillings, and then "within six seconds drinks ice water at 32° F.," getting this also carefully in contact with his gold fillings, one can only regret his want of common sense and consideration for his own feelings. His example is not likely to be followed generally.

He states that amalgam is acted on by the liquids of the mouth sufficiently to fill up small cracks between tooth and filling and to become *cemented to the tooth.* May I ask if any operator has ever seen such an effect as this in one single case in the course of his practice?

He states that "*all* silver amalgams are very soft, shrink considerably, and lose relatively easily their mercury." If he will take pure precipitated silver and mix it with no more mercury than is absolutely required to make a hard plug, packing it in a glass tube which is sealed so firmly as to prevent lifting, he will find in a few days that the expansion has burst this tube unless it is excessively strong, and that the plug is so hard as to be cut out only with great labor. It is certainly hard enough to stand twenty years on the grinding surface of a molar.

Again, he says, "the mercury evaporates from platinum as if no combination had taken place." If he had said *when* no combination takes place, he would have been correct; mercury and platinum combine with difficulty, and only under peculiar conditions, and it is evident he has not succeeded in obtaining this combination.

He is "at a loss to say why makers put gold or platinum in their amalgams if it is not to give them some nice taking name." If he will take an alloy which has platinum *in proper combination* and the same without platinum, he will find that the former sets hard in about one-fourth the time, that it is much harder, and that it is far less liable to alter in form or ball up after hardening. A mixture of platinum with other metals without proper combination is of no value, and is apparently what he has experimented with. Platinum cannot be got in combination by the processes usually published. The use of gold is to make an amalgam cleaner and pleasanter in the hand, and its use, except to a limited extent, is to be condemned, as it reduces the setting power seriously. He says oxyphosphates "resist" far more than oxychlorides. If he will take a first rate phosphate of zinc cement and a first-rate oxychloride of zinc, he will find that the resistance to all solvents is wonderfully similar, the difference is detected only by careful experiment, and, if anything, on the average is in favor of the oxychloride. It is no test to take a good sample of one and a bad one of the other. The phosphate cements have the advantage of being harder and less liable to be damaged by moisture, but so far as actual permanence is concerned, in the hands of a good operator there is no choice. The phosphate cements are at present in the fashion, and are more generally successful in the hands of careless, slovenly operators, but with proper manipulation and proper care the oxychlorides are, when good, quite as permanent and have the advantage of being far less opaque. The careless operator is still a large element, and he has to be studied by the makers of filling materials; for him the phosphates are a blessing, as his carelessness is less liable to cause failures with this than with any other white filling.

When a writer begins by acknowledging that his task is too difficult for him, and then goes on to prove this statement most thoroughly, his contribution is liable to do much more harm than good.—*British Journal of Dental Science.*

Apropos to the above, we print the subjoined extract from an editorial in the same number of the *Journal:*

In the last number of this journal we republished from an American source, an article on "The Chemical and Physical Effects of Fillings upon Teeth," which we fully expected would elicit some replies. And in this we have not been disappointed, for it has called forth an answer from one who is, in this country at least, the best qualified to undertake the task. Mr. Mayr is a chemist and physicist of some reputation in his own land, but he is careful to tell us that his practical experience of Dentistry is very limited; Mr. Fletcher, on the other hand, besides being an accomplished chemist, has the further advantage of being a practical Dentist of no mean skill; it is not to be wondered at, then, that he has little difficulty in disposing of Mr. Mayr's rather surprising statements.

But it is not with these that we wish to concern ourselves just now; we wish rather to consider in what spirit suggestions from an outsider should be received. It seems to us that this should depend entirely on the spirit in which they are made. When a man puts himself forward and dogmatises blindly on a subject about which he knows little or nothing, he deserves to be put down or, as it is generally termed, snubbed. But if, as in this case, the speaker states honestly, I don't know anything about Dentistry, but I do know something of chemistry, and I will tell you how my knowledge leads me to view some of your processes which are governed by the laws of chemistry, we think that man deserves different treatment, and from this point of view we think Mr. Fletcher is rather too severe on his opponent.

We would especially dissent from Mr. Fletcher's opinion that such a paper as this is likely to do more harm than good. In our opinion nothing is more improving than the criticisms of an educated layman. We are all far too prone to adopt practices empirically and without properly thinking out and weighing the steps by which we have arrived at a given conclusion. Few can boast of the combination of theoretical with practical knowledge which Mr. Fletcher himself possesses, and it needs some such stimulus as our chemical friend supplies to rouse us to give an account of the faith that is in us.—*Dental Advertiser.*

